# Grip Strength and Its Relationship to Police Recruit Task Performance and Injury Risk: A Retrospective Cohort Study

**DOI:** 10.3390/ijerph14080941

**Published:** 2017-08-21

**Authors:** Robin Orr, Rodney Pope, Michael Stierli, Benjamin Hinton

**Affiliations:** 1 Tactical Research Unit, Bond University, Robina, QLD 4213, Australia; rpope@bond.edu.au; 2 New South Wales Police Force, Surry Hills, NSW 2010, Australia; stie1mic@police.nsw.gov.au (M.S.); hint1ben@police.nsw.gov.au (B.H.)

**Keywords:** assessment, hand strength, marksmanship, law enforcement, tactical, task performance

## Abstract

Suitable grip strength is a police occupational requirement. The aim of this study was to investigate the association between grip strength, task performance and injury risk in a police population. Retrospective data of police recruits (n = 169) who had undergone basic recruit training were provided, including handgrip strength results, occupational task performance measures (consisting of police task simulations [SIM], tactical options [TACOPS] and marksmanship assessments) and injury records. Left hand grip strength (41.91 ± 8.29 kg) measures showed a stronger correlation than right hand grip strength (42.15 ± 8.53 kg) with all outcome measures. Recruits whose grip strength scores were lower were significantly more susceptible to failing the TACOPS occupational task assessment than those with greater grip strength scores, with significant (*p* ≤ 0.003) weak to moderate, positive correlations found between grip strength and TACOPS performance. A significant (*p* < 0.0001) correlation was found between grip strength, most notably of the left hand, and marksmanship performance, with those performing better in marksmanship having higher grip strength. Left hand grip strength was significantly associated with injury risk (*r* = −0.181, *p* = 0.018) but right hand grip strength was not. A positive association exists between handgrip strength and police recruit task performance (notably TACOPS and marksmanship) with recruits who scored poorly on grip strength being at greatest risk of occupational assessment task failure.

## 1. Introduction

Police officers have a primarily sedentary occupational workload which can be punctuated by periods of highly physically demanding tasks [1,2]. These dynamic tasks, which can be performed with minimal notice and maximal effort, include running, jumping, crawling, balancing, climbing, lifting, carrying, pushing, pulling, fighting, dragging and restraining a suspect, and are often performed in unpredictable environments [3,4]. These physically demanding tasks and the circumstances in which they are performed punctuate the essential need for police officers to sustain a suitable level of fitness in order to achieve occupational task success. 

Apart from the aforementioned and obviously physical tasks, there are other tasks that may not necessarily be typically considered as physically demanding but may still require elements of physical capability. Tasks such as the control of a firearm and the ability of an officer to fire the weapon with optimal precision serve as examples. Several studies have identified that the estimated proportion of police rounds missing their targets can range from between 52 per cent and 65 per cent [5,6]. Therefore, the ability of police officers to perform these tasks effectively reduces the likelihood of not only endangering not only the individual, but also fellow officers and, at times, the general public. Several explanations have been offered for poor police marksmanship, such as the lack of valid training in real-life circumstances and neurophysiological limitations which could potentially limit a police officers’ ability to enhance their marksmanship [7,8]. Considering this, several studies have investigated the relationship between grip strength and handgun marksmanship in police recruits [5,9,10,11]. While the results have been mixed, with studies finding either associations [1,5,9] or no associations [11] between grip strength and marksmanship, on balance this research does suggest a positive relationship between measures of grip strength and marksmanship. Nevertheless, further research is needed to strengthen the evidence regarding relationships between grip strength and marksmanship, as well as potentially the relationship between grip strength and the performance of other tasks performed by police officers.

A requirement for police officers to have suitable grip strength for task performance is not surprising given that grip strength and grasp actions are required in tasks that range from apprehension, grappling and victim rescue, to the use of specialty equipment and, as noted above, use of firearms [6]. For these reasons, amongst others, handgrip and grasp measures have been used in tactical populations [11,12,13], with grip strength specifically used as an assessment measure during qualification and recruitment [1,12,14].

Considering the potential relationships between grip strength and task performance and its current use in tactical populations, grip strength assessment may also provide some utility as a predictor of injury risk during training. Research suggests that new trainees are at a higher risk of injury than qualified tactical personnel, with less fit personnel in particular being at greater risk of injury [15]. As an example, a study by Pope, Herbert, Kirwan and Graham [16] found that the least fit recruits were 14 times more likely to be injured than recruits who scored high on a 20 Progressive Shuttle Run Test, findings that have been supported by other literature [17,18,19]. Given that injury is a strong predictor of recruit attrition and that it can cost a police department more than AUD$ 85,000 to recruit and train a replacement police officer [20], ways of predicting and preventing injury in new recruits are important. 

With the potential for grip strength to be used as more than just a measure of fitness, further investigation into its use as a predictor of task performance and risk of injury is required, specifically in the population of interest; in this instance, law enforcement. Therefore, the aim of this study was to evaluate the predictive validity of grip strength in police recruits for predicting police task performance and to identify relationships between grip strength and potential for injury during training.

## 2. Materials and Methods

The study used a retrospective cohort design, involving data previously collected by Police Physical Training Instructors. The retrospective data was provided, in non-identifiable form, for police recruits who underwent Session 2 of their general police recruit training (12 weeks) in the calendar year of 2013, at a police college in Australia. Session 2 of training was selected for this program of research as it is during this session that key assessments of performance on occupational tasks are conducted. Ethics approval for the study was provided by the Bond University Human Research Ethics Committee (RO 1898).

### 2.1. Study Population

One hundred and sixty-nine police recruits constituted the sample for this study. The recruits had been undergoing full-time training at the police college at the time their data were collected. No demographic information on these recruits was available, however all police recruits are required to meet entry requirements for age (a minimum of 18 years and 4 months of age), to have completed a health clearance from a General Practitioner and to have had a full medical assessment completed by an external provider. The lack of detailed demographic data is not uncommon in tactical environments [21,22], given the requirement to ensure the identities of participants are protected. Inclusion criteria were: (a) the recruit had attended Session 2 of police recruit training at the time of data collection; (b) the recruit had not attempted Session 2 previously; and (c) the recruit was able to complete the grip strength assessment and all occupational task assessments. The exclusion criterion for this study was recruits who were suffering an injury at the time of initial fitness assessment.

### 2.2. Measurements

The grip strength assessments were performed by all recruits in a single session at the commencement of Session 2 of police recruit training (Week 1). The grip strength assessments were conducted in accordance with approved NSW police physical training protocols and the assessors were unaware of the research. The occupational task performance assessments of a simulation task (SIM), tactical options assessment (TACOPS) and marksmanship were conducted as part of the standard recruit training program. Injury data were collected during the program by police college staff and provided to the research team after the conclusion of training, along with grip strength, SIM, TACOPS and marksmanship data.

#### 2.2.1. Grip Strength

Using a handgrip dynamometer (TTM Original Dynamometer, Tokyo, Japan), maximum grip strength of right and left hands was measured to the nearest kilogram. The protocol detailed by Dortkamp [23] was followed, whereby subjects were instructed to keep their arms by their side, without touching the body, throughout the measurement, as they squeezed the dynamometer. Two recordings were taken for each hand but if a subject had a difference of greater than 5% between the first and second measurements, then a third trial was undertaken. The highest measure of the two or three attempts was recorded for each hand. At the time the testing was undertaken, recruits, regardless of age or gender, were expected to achieve a score of 30 kg for both hands.

#### 2.2.2. Task Performance Measures

The selected occupational task performance measures were derived from the assessable occupational task requirements of new police recruits. These occupational task performance measures, considered to be representative of those required of serving police officers, were completed as part of standard recruit training. These measures are as follows:

*Simulation Task (SIM):* Police recruits were required to perform simulated tasks including basic tactics of defence (e.g. restraining belligerent assailants and handcuffing in a scenario where an officer dresses in protective equipment or “Redman” suit to allow practical application of techniques) and the use of simulated munitions. Police instructors scored the recruits as pass or fail based on their technique and performance within the SIM.

*Tactical Options Assessments (TACOPS):* Police recruits were required to respond to given scenarios, specific to police operations, and employ the most appropriate tactical options to resolve the situation with as minimal force as possible to neutralize the threat. Police instructors scored the recruits as pass or fail based on the tactical options they selected and their application of these options.

*Marksmanship:* The marksmanship assessment was conducted later in the police recruit training program. The assessment required recruits to engage a standard Z-4 police target with a Glock self-loading pistol firing allocated police. 40 calibre Smith and Wesson ammunition. A total of 30 scoring rounds over several serials were conducted. A pass score of 80 points was required with points awarded depending on figure strike zone with zero points awarded for a miss and one to four points per round on target. All participants stood in an isosceles stance (in which the officer stands square on to the target with feet shoulder width apart and toes level) with both arms fully extended towards the target and the pistol gripped firmly in both hands.

### 2.3. Injury

Over the course of the training period, injury data were collected as per standard police college protocol. Injuries were documented using a standard Accident and Incident Form, in accordance with normal police processes. Injury was defined as physical damage to the neuro-musculoskeletal system of the body. Determination of injury was made by the treating medical staff who were unaware of the research. Upon cohort graduation, the research team was provided with the injury data aligned with the recruit’s other measures. Injury was denoted with a “1” score and no injury with a “0” score. Due to data access limitations, no information regarding injury type, site or severity were available.

### 2.4. Data Analysis

The non-identifiable data provided by the police force were entered into Microsoft Excel and checked. The data were then imported into SPSS. Following descriptive analysis, Spearman’s correlation analyses with tie correction ([24], pp. 1371–1373) were used to establish relationships between measured grip strength and all ordinal outcome measures (including task performance measures of SIM and TACOPS, and injury status), since statistical assumptions for point-biserial or biserial correlation analyses and other parametric tests of association could not be met ([24], pp. 1324–1329). In the Spearman’s correlation analyses, the dichotomous injury status or pass/fail variable was considered ordinal since a value of “0” indicated no injury or pass had occurred and a value of ‘1’ indicated at least one but possibly more than one injury had occurred or that a fail had occurred, though the extent to which the officer had passed might be variable. Pearson’s correlation analyses were used to assess relationships between measured grip strengths of right and left hands and between grip strength of each hand and pistol marksmanship scores. A linear regression analysis was performed to assess the relationship between marksmanship and grip strength, using each of left and right hand grip strength separately due to collinearity concerns, and to produce a regression equation for predicting shooting scores—the latter selected based on the regression model (using right or left hand grip) that explained the greatest proportion of variance in marksmanship scores. Differences between right and left hand were also determined (Right–Left). Independent samples t-tests were conducted to identify differences in mean grip strengths between “injury” and “no injury” groups as well as between “pass” and “fail” groups based on all other performance measures. To allow for graphical representation of the actual (rather than “fitted”) relationships between marksmanship scores and handgrip strength, handgrip strength was categorised using 5 kg bin groupings, except at the upper and lower extremes of the range of grip strengths, where officers were grouped to ensure at least five were present in each grouping, to provide a reasonable basis for calculation of key statistics for the grouping. Boxplots were then developed to depict the actual relationships between grip strength and marksmanship scores. Statistical analyses were conducted using the Statistical Package for the Social Sciences (SPSS, version 23), with the alpha level set at .05, *a priori*.

## 3. Results

Of the 169 participants (all of whom were right hand dominant) for which data were available, mean grip strength scores were 42.15 kg (±8.29, range 25 to 67 kg) for the right hand and 41.91 kg (±8.53, range 24 to 60 kg) for the left hand. Comparisons of the right and left hand grip strengths showed a mean difference of 0.24 kg (±4.89, range –15 to 12 kg). The correlation between right and left hand grip strengths was both strong and significant (*r* = 0.831, *p* < 0.001).

Only 5.3% of recruit participants (n = 9) did not reach the minimum required grip strength of 30 kg in at least one hand and among these, 77.8% (n = 7) failed at least one assessment or were injured. One participant failed to meet the grip strength requirement in either hand and this participant failed both SIM and TACOPS.

During task performance measures, 55.0% (n = 93) of participants passed the SIM, 58.6% (n = 99) of participants passed TACOPS and 84.0% (n = 142) of participants passed the marksmanship test on their first attempts. An injury was reported by 25.4% (n = 43) of participants. Descriptive statistics indicating mean grip strength in each hand by outcome status (pass/fail or injury/no injury) for each of the four occupational performance measures are shown in Table 1.

**Figure 1:**
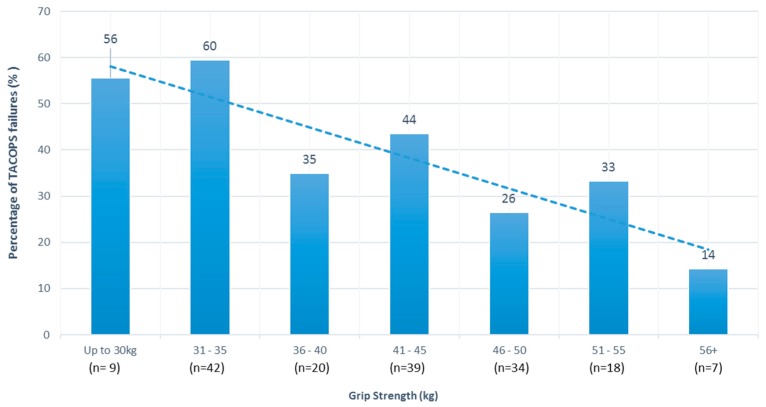
Percentage of recruits failing TACOPS by right handgrip strength level in bin groupings.

Despite the higher mean grip strength scores in Table 1 for those who passed the SIM test, there was no significant association observed between grip strength scores and SIM pass rates (Table 2). Likewise, there were no significant differences observed between SIM pass and fail groups in mean grip strength scores (Table 1). The levels of association between grip strength scores and TACOPS results were positive and statistically significant (right hand *r* = 0.227, *p* = 0.003: left hand *r* = 0.269, *p* < 0.0001), indicating that participants with higher grip strength were more likely to be in the pass group for the TACOPS assessment. When the data were split by grip strength groupings, it was evident that likelihood of passing increased with increased grip strength. There were significant differences in mean grip strength for both right (*p* = 0.004) and left (*p* < 0.001) hand between participants who “passed” and those who “failed” TACOPS (Table 1). The relationship is best shown in a chart such as Figure 1, where it is evident that only 44% of recruits with a grip strength of less than 30 kg in the right hand passed the TACOPS, compared to a pass rate of 86% for those recruits with a grip strength of greater than 55 kg. Cumulatively, up to a grip strength of 35 kg in the right hand, pass rates averaged 41% on TACOPS, increasing to 73% in recruits who scored over 45 kg on right hand grip strength.

**Figure 2:**
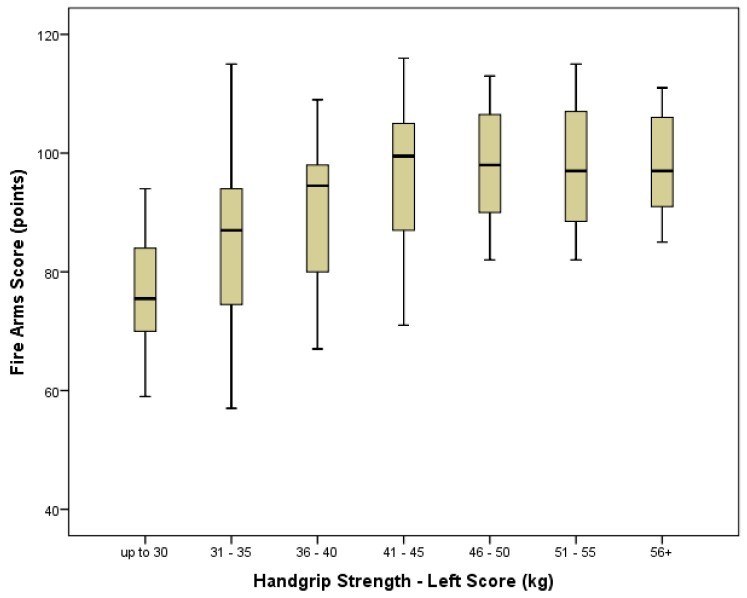
Boxplot of relationship between left hand grip strength and marksmanship score displayed in bin groupings.

**Figure 3:**
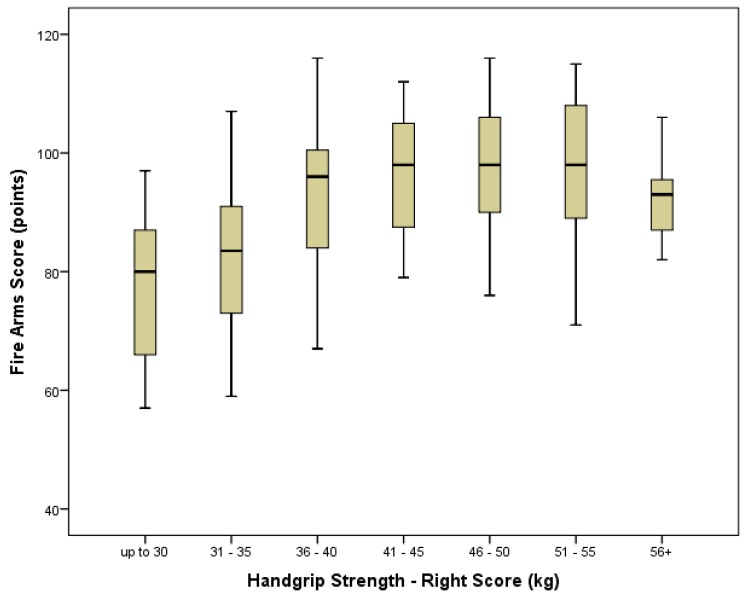
Boxplot of relationship between right hand grip strength and marksmanship score displayed in bin groupings. The horizontal line in the center of the box represents the median with the top and the bottom of the box representing the 75th and the 25th percentiles respectively. The crossed vertical lines above and below the box represent the maximum and minimum values with extreme outliers removed.

Grip strength score also significantly predicted marksmanship score (right hand *r* = 0.398, *p* < 0.0001: left hand *r* = 0.475, *p* < 0.0001) (Figure 2 and Figure 3) with significant differences in both mean right (*p* < 0.001) and left (*p* < 0.001) hand grip strength scores between those who “passed” and those who “failed” their marksmanship assessment. The optimal regression equation for predicting marksmanship scores from grip strength was: Marksmanship score = 56.1 + 0.843 × (left hand grip strength [kg]). Levels of association between grip strength score and injury status were negative, weak, and non-significant for the right hand (*r* = −0.137, *p* = 0.076). However, the level of association reached significance for the left hand (*r* = −0.181, *p* = 0.018) (Table 2). Considering this, while there were no significant differences in mean right hand grip strength (*p* = 0.065) scores between those suffering an injury and those reporting no injury, there was a significant difference in mean left hand scores (*p* = 0.019).

## 4. Discussion

The present study investigated the associations between grip strength and the occupational task performance measures of SIM, TACOPS and marksmanship, as well as injury risk, in police recruits. With a mean grip strength of 42.15 kg (±8.29 kg) recorded for the right hand and 41.91 kg (±8.53 kg) for the left hand, the results showed that only 5.3% of recruit participants (n = 9) did not reach the minimum required grip strength of over 30 kg. However, from among the recruit participants, 45% (n = 73) failed the SIM, 41.4% (n = 70) failed TACOPS, 16% (n = 27) failed the marksmanship assessment and 25.4% (n = 43) suffered an injury. TACOPS and marksmanship results were significantly and positively associated with grip strength performance, with better scores on TACOPS and marksmanship indicating increasing task completion success. Injury risk was negatively and weakly correlated with left hand grip strength but not right hand grip strength. Conversely, no significant association was found between grip strength and SIM pass rates.

### 4.1. Normative Comparisons

Previous studies have provided grip strength normative data and found that mean grip strength was greater among men than women in the general population [25,26]. Perna, Coa, Troiano, Lawman, Wang, et al. [26] used a combined grip strength score (right plus left grip strength) and found mean scores of 98.2 kg and 61.9 kg, for men and women respectively. In the general Australian population, Egger, Champion and Boulton [27] suggest that the average male grip strength ranges from 46 kg (left hand) to 50 kg (right hand) and the average female grip strength ranges from 25 kg (left hand) to 29 kg (right hand). In a police specific population, Dawes, Orr, Flores, Lockie, Kornhauser and Holmes [28] found that female police officers presented with a mean grip strength of 37.875 ± 5.34 kg and male officers with a significantly greater mean grip strength of 55.04 ± 7.77 kg—a range that brackets the findings of this study, which included female and male data combined.

Acknowledging these findings of differences between sexes, previous research has suggested that a sex neutral approach should be advocated where occupational task requirements are sex neutral, focusing either on occupational injury risk [16] or absolute task performance [29] rather than general fitness and health. This may be particularly important for law enforcement populations, where officers must be able to complete tasks regardless of sex and may be required to interact with people of either sex (for example, a female officer may need to restrain a male offender). The results gathered in this study, which did not consider sex of the police recruits, found that mean grip strength scores for the left and right hand of the police recruits in this cohort were slightly lower than the mean grip strength of the general male population (left lower by 4.09 kg: right lower by 7.85 kg), while right and left hand grip strengths were strongly correlated.

Interestingly, the findings of this study differed from that of Luna-Heredia, Martín-Peña, and Ruiz-Galiana [25]. In the study by Luna-Heredia et al. [25], while sex differences were still reported, there were significant differences between dominant and non-dominant hands, such that peak grip strength was 50.9 kg in the dominant hand and 41.2 kg in the non-dominant hand for males and 28.2 kg in the dominant hand and 23.5 kg in the non-dominant hand for females. In contrast, this study found almost identical mean scores for the right (dominant in this population) and left hand (mean difference of 0.24 kg).

### 4.2. Grip Strength and Occupational Task Performance (SIM and TACOPS)

The results of this study indicate that recruits whose grip strength scores were lower were significantly more susceptible to failing the TACOPS occupational task assessment than those with greater grip strength scores. No such significant relationship was observed for the SIM task, despite mean grip strength scores being marginally lower in recruits who failed the SIM task than in those who passed the SIM task.

Figure 1 demonstrates the utility of grip strength for predicting TACOPS assessment results, even though the relationship was only weak (right hand *r* = 0.227: left hand *r* = 0.269). Since TACOPS is an occupational assessment of importance throughout a police officer’s career and indicates a police officer’s ability to maintain operational safety, the relationship between grip strength and TACOPS outcomes, when combined with the repeated need for a police officer to demonstrate the capacities assessed by the poor grip strength, will have a cumulative effect on risk. Every episode of activity of the police officer, throughout their career, which requires capacities assessed by TACOPS, will be impaired by the lack of grip strength, with associated risks of an adverse outcome from such activities accumulating over time. This phenomenon of differential risk accrual in different risk groups, over repeated exposures to a physical activity event over time, is discussed in detail by Pope [30]. The results of this study are not surprising given that several studies support the association of increased grip strength with improved occupational performance in other tactical populations [31,32,33,34]. 

The evidence from this study combines with evidence from previous research to comprise an increasing body of evidence indicating a positive association between performance in occupational tasks of tactical populations, like police, military and fire fighting populations, and grip strength. However, there has been limited evidence to date to indicate the minimum required grip strength necessary to optimally perform occupational tasks essential to these populations. This difficulty is highlighted again by this research, where there were officers (albeit relatively few) who scored poorly yet passed their assessments, or scored well, yet failed their assessments. This can be explained by the fact that performance on occupational tasks is determined by multiple factors, and grip strength is just one of those, albeit important based on the current findings. On this basis, employment of specific grip strength standards needs to consider what level of capability risk an organisation is willing to accept, lower standards will increase risks of adverse outcomes occurring during police activities, and higher standards may mean that potential successful candidates are excluded by the screening process.

### 4.3. Grip Strength and Marksmanship

This study found a significant correlation between grip strength, most notably of the left hand, and marksmanship performance. As with the SIM and TACOPS tasks, recruits with a lower grip strength were found to perform more poorly in marksmanship than those with a higher grip strength. As demonstrated in Figure 2 and Figure 3, recruits with grip strength of over 35 kg (either hand) were generally more likely to achieve a score of over 80 points and pass their marksmanship assessment compared to those with lower grip strength. Interestingly, while score improvement tended to plateau in the grip strength range of 41^+^ kg, there was a slight downward trend in marksmanship scores for those with grip strengths over 60 kg in the right hand (left hand scores did not reach higher than 60 kg), suggesting a potential limiting impact on marksmanship of a very high grip strength.

Supporting the findings of our study, other studies [1,9,35,36] have likewise found that marksmanship scores on the qualifying firearms test were significantly correlated to maximal grip strength. These studies included marksmanship tasks with a Glock pistol [9,35,36], Beretta pistol [1,35], or Smith and Wesson revolver [36]. Of particular note, the correlation between maximal grip strength and marksmanship scores in the study of Anderson and Plecas [1] was remarkably similar to the results of this study (Anderson, *r* = 0.38, *p* < 0.05; this study *r* = 0.398, *p* < 0.0001).

As a point of note, research [1,5,8,37,38,39] also suggests that grip strength by itself is just one of several factors that determine marksmanship scores, with significant contributions also from static upper body strength and endurance, physiological factors (such as nerve impulse speed, muscle response time, average heart rate, and maximal heart rate) as well as the level of training received by the officer. Considering this variety of influencing factors, it should be noted that all participants in this study were participating in a standardised trainee program and had therefore completed similar weapons training over the 12-week instructional period.

### 4.4. Grip Strength and Injury

Currently, many studies suggest that good grip strength is associated with less disability as adults age [26]. Considering this, the findings of this study indicated only a weak association between grip strength and risk of injury in police recruits. Consistent with the findings of our study, a study by Dale, Addison and Lester [40] explored grip strength associations in construction, office and service workers. Their study found no consistent association between grip strength and health outcomes during a three-year follow-up of newly employed workers. They also concluded that past musculoskeletal disorders were the strongest predictor of future symptoms or work disability [40]. On this basis, while the utility of grip strength as a means of predicting injury is currently in doubt and requires further research, this outcome measure may be useful in return-to-training planning for police officer recruits undergoing treatment for upper limb injuries, given its utility for predicting TACOPS and marksmanship performance, as measures of occupational readiness.

### 4.5. Limitations

This study had several limitations. The key limitation of the study was the lack of detail within the provided data. Firstly, no demographic data for the participants were provided except that participants met the minimum age requirement to undergo police training. This lack of detailed demographic data limited comparisons and ability to publish age, sex and morphological specific normative data and as such further research is required when considering the use of these findings as an occupational requirement. Secondly, there was a lack of detail provided in the dataset regarding the nature of injury, i.e., type, bodily site and severity. More detailed data would have been of benefit to provide a more detailed profile of the injuries present, to enable estimation of training time loss and the recovery costs. Finally, the sample size was limited when it came to assessing the utility of grip strength measures for predicting injury risk, since injury is a relatively rare event, and further investigation of this relationship in larger samples of police recruits is warranted. Likewise, all participants were right handed and as such, there may be some variability in these findings (for example, the differences found between right and left hand grip and their relationships to the variables) when applied to left handed officers.

## 5. Conclusions

The results of this study suggest that an association exists between handgrip strength and police task performance, as measured by TACOPS and marksmanship, particularly. The utility of grip strength as a measure of injury risk requires more research in larger samples with more detailed injury-specific data including, for example, types, anatomical sites, and severities of the injuries sustained. In general, it appears that recruits with poorer grip strength, in the left hand more than the right, may be at a higher risk of specialised police task failure when compared to those recruits with higher grip strength results. On this basis, optimizing the grip strength of police recruits before they enlist, whilst under training and, if needed, during any rehabilitation, would be of importance.

## Figures and Tables

**Table 1:** Mean ± SD for grip strength by occupational performance outcomes and injury status.

		Mean Grip Left (kg)	Mean Grip Right (kg)
**Population**	(n = 169)	41.91 ± 8.53	42.15 ± 8.29
**SIM**	Pass (n = 93)	42.98 ± 8.81	42.78 ± 8.20
	Fail (n = 76)	40.59 ± 8.03	41.38 ± 8.39
**TACOPS**	Pass (n = 99)	43.82 ± 8.72 *	43.68 ± 8.36 *
	Fail (n = 70)	39.20 ± 7.51	40.00 ± 7.74
**Marksmanship**	Pass (n = 142)	43.53 ± 8.10 *	43.45 ± 8.02 *
	Fail (n = 26)	33.37 ± 4.87	35.33 ± 6.08
**Injury Status**	No Injury (n = 126)	42.80 ± 8.23 **	42.84 ± 8.13
	Injured (n = 43)	39.28 ± 8.92	40.14 ± 8.50

Significant difference between pass/fail or injured/no injury groups, * *p* < 0.01, ** *p* < 0.05. SIM = Police task simulations, TACOPS = Tactical options assessment.

**Table 2:** Correlations between grip strength and performance measures.

Measure	SIM *	TACOPS *	Marksmanship **	Injury Status *
Grip Right	*r* = 0.095 (*p* = 0.217)	*r* = 0.227 (*p* = 0.003)	*r* = 0.398 (*p* < 0.0001)	*r* = −0.137 (*p* = 0.076)
Grip Left	*r* = 0.135 (*p* = 0.081)	*r* = 0.269 (*p* < 0.0001)	*r* = 0.475 (*p* < 0.0001)	*r* = −0.181 (*p* = 0.018)

* Spearman’s Correlation, ** Pearson’s Correlation.
